# Sex-Related Factors in Cardiovascular Complications Associated to COVID-19

**DOI:** 10.3390/biom12010021

**Published:** 2021-12-24

**Authors:** Francesca Megiorni, Paola Pontecorvi, Giulia Gerini, Eleni Anastasiadou, Cinzia Marchese, Simona Ceccarelli

**Affiliations:** Department of Experimental Medicine, “Sapienza” University of Rome, Viale Regina Elena 324, 00161 Rome, Italy; francesca.megiorni@uniroma1.it (F.M.); paola.pontecorvi@uniroma1.it (P.P.); giulia.gerini@uniroma1.it (G.G.); eleni.anastasiadou@uniroma1.it (E.A.); cinzia.marchese@uniroma1.it (C.M.)

**Keywords:** SARS-CoV-2, COVID-19, cardiovascular diseases, sex, gender, biomarkers

## Abstract

Coronavirus disease 2019 (COVID-19), the pandemic infection caused by the severe acute respiratory syndrome coronavirus 2 (SARS-CoV-2), presents with an extremely heterogeneous spectrum of symptoms and signs. The clinical manifestations seem to be correlated with disease severity. COVID-19 susceptibility and mortality show a significant sex imbalance, with men being more prone to infection and showing a higher rate of hospitalization and mortality compared to women. Such variability can be ascribed to both sex-related biological factors and gender-related behavioral cues. This review will discuss the potential mechanisms accounting for sex/gender influence in vulnerability to COVID-19. Cardiovascular diseases play a central role in determining COVID-19 outcome, whether they are pre-existent or arose upon infection. We will pay particular attention to the impact of sex and gender on cardiovascular manifestations related to COVID-19. Finally, we will discuss the sex-dependent variability in some biomarkers for the evaluation of COVID-19 infection and prognosis. The aim of this work is to highlight the significance of gendered medicine in setting up personalized programs for COVID-19 prevention, clinical evaluation and treatment.

## 1. Introduction

Coronavirus disease 2019 (COVID-19) is a pandemic infection caused by the novel severe acute respiratory syndrome coronavirus 2 (SARS-CoV-2). COVID-19 infection developed in December 2019 [1], and then rapidly spread out across the rest of the world, earning the definition of “pandemic” from the World Health Organization in March 2020. As of 5 October 2021, there were more than 237 million cases of COVID-19 and 4.8 million deaths, with a case fatality rate of 2.0% [2]. The effects of SARS-CoV-2 infection are quite variable, and COVID-19 presents with a wide spectrum of symptoms and signs, ranging from no symptoms at all (asymptomatic subjects) to severe respiratory and systemic ones [3,4,5,6]. A significant number of symptomatic patients require hospitalization, and approximately 3% of them have a fatal outcome [7,8,9]. More than half of symptomatic patients also develop a post-acute syndrome designated “long COVID”, characterized by persistent effects or long-term complications occurring beyond 4 weeks from the onset of symptoms and lasting from weeks to months [10].

The development of symptoms seems to be correlated with more severe disease and poor prognosis. For this reason, several studies aimed to identify the determinants for the development of symptomatic disease, in order to stratify patients based on their susceptibility and to target preventive and therapeutic practices. In several reports on COVID-19 patients, old age, male sex, and the presence of some comorbidities appear to correlate with developing symptomatic disease and with bad prognosis, indicating that such patients should be given much more attention in prevention, treatment and follow-up [11,12,13,14,15]. Older age can be identified as a significant risk factor for developing symptomatic COVID-19, due to the natural decline of the immune system with age, and to the more probable presence of comorbidities [16]. In the context of underlying conditions determining COVID-19 outcome, diabetes mellitus shows particular relevance due to its association with immune system depression that makes those patients more susceptible to any infectious diseases, including COVID-19 [17,18].

**Figure 1:**
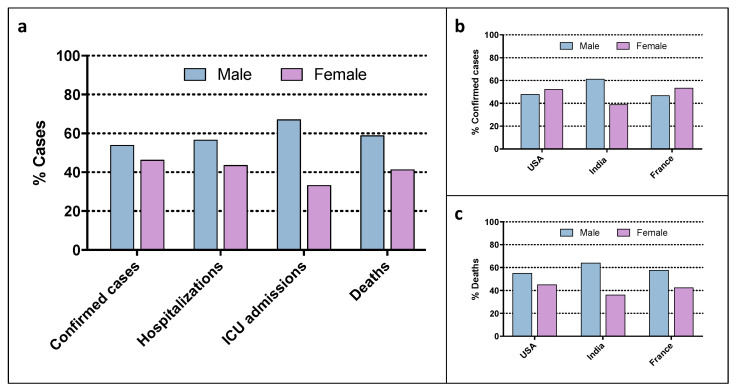
(**a**) Sex-stratified incidence and outcome of patients who tested positive for COVID-19 globally; (**b**,**c**) sex-stratified incidence (**b**) and death (**c**) of patients who tested positive for COVID-19 in the three countries with most confirmed COVID-19 cases. ICU, intensive care unit. Source: the COVID-19 Sex-Disaggregated Data Tracker, as of 26 October 2021.

Reports of COVID-19 symptoms and mortality from most countries show that a significantly higher proportion of men are being hospitalized and dying from COVID-19 compared to women [19,20,21]. Other studies pointed out a sex imbalance not only in the development of symptoms or in the severity of the prognosis, but also in the susceptibility to SARS-CoV-2 infection, with the male sex being the most vulnerable. Globally, the mean percentages of affected men and women indicate a difference in the number of cases (53.8 vs. 46.2%), and also a significant sex gap in hospitalization rates (56.5 vs. 46.5%), intensive care unit (ICU) admissions (66.9 vs. 33.1%) and mortality (58.8 vs. 41.2%) (Figure 1a) [22]. The analysis of sex-disaggregated data in the three countries with a higher number of COVID-19 cases (USA, India and France) reveals the same trend of male prevalence for patients’ mortality (Figure 1c), while COVID-19 incidence showed a significant sex difference only among Indian patients (Figure 1b). 

To understand the possible explanations for this difference among countries, it is important to specify that biological sex and gender are two distinct concepts that both can be involved in COVID-19 infection and outcome. In fact, sex is determined by biological attributes of females and males, and it influences the immunological and hormonal profiles that may be important in responding to an infection and so in determining the clinical outcome in patients with COVID-19 [23]. Gender depends on socially constructed roles, behaviors, expressions and identities, and it could play a critical role in the exposure to the virus [23]. In this light, gender factors related to discrimination towards the female population in Indian society might account for the differential COVID-19 incidence between the two sexes. 

We discuss below the potential mechanisms accounting for sex/gender influence in vulnerability to COVID-19, ranging from biological factors, such as the differential expression of genes involved in virus infection and in immune response, to behavioral issues, related to women’s better attention to health issues. In particular, we will pay attention to the impact of sex and gender on cardiovascular manifestations related to COVID-19. 

## 2. Sex and Gender Factors in COVID-19 Infection and Outcome

The sex differences in SARS-CoV-2 infection incidence and mortality highlighted by literature [21,24] can arise from genetic factors that influence virus entry, but also from sex determinants regarding the immune system response to viral infection or to treatment, as well as gender-related issues influencing viral exposure.

### 2.1. Genetic Factors

The male–female disparity in COVID-19 infection and outcomes may derive from mere genetic factors. The inactivation of the X chromosome (XCI) is particularly relevant in the discussion concerning the immune response against SARS-CoV-2. In fact, females have two X chromosomes, containing several genes important for innate and adaptive immunity, including those involved in the Toll-Like Receptors pathway [25]. The inactivation of one of the two X chromosomes ensures the right gene dosage. However, it is known that several genes can escape from the inactivation of the X chromosome, this resulting in biallelic gene expression in females. Expression of X-inactive specific transcript (XIST) RNA has been observed in both B and T cells, with a double dosage of CXCR3, TLR7 and CD40L gene products [25,26]. Moreover, this mechanism may lead to a higher proportion of cells expressing functionally more advantageous alleles [25,27]. Indeed, X-chromosome skewing and cellular mosaicism may enable females to better react to immune challenges, particularly viral infections such as SARS-CoV-2. In addition, the X chromosome contains a huge number of microRNAs compared to the Y chromosome, with many of them targeting immuno-suppressive genes such as FOXP3, CTLA4, CBL and SOCS [28]. This is another fact that may help to explain the sex bias in immune responses. Additionally, the gene coding for angiotensin-converting enzyme 2 (ACE2), a protein that mediates SARS-CoV and SARS-CoV-2 entry into human cells [29,30], is located on the X chromosome. The expression of ACE2 seems to be higher in females compared to males, but it is still not clear if this depends on XCI escape or on the action of estrogens [24]. 

### 2.2. Sex Hormones

One of the main reasons for the gap between males and females in COVID-19 pathogenesis and infection could be the different modulation of cellular receptor and coreceptors used by SARS-CoV-2 to enter the human host cells. Animal studies showed that ACE2 is retained in females more than males, even upon aging and in the presence of chronic disease [31]. This suggests that gonadal hormones rather than sex chromosomes may be involved in these sex differences [32]. In fact, ACE2 gene expression is regulated by estrogens, whereas the TMPRSS2 gene, encoding for an important priming enzyme required during viral entry, is controlled by an androgen-responsive promoter [33]. Besides its role as a receptor for SARS-CoV-2 entry into the cells, ACE2 is also a key modulator of the renin–angiotensin system (RAS), a signaling pathway involved in the regulation of vascular function. ACE2 converts angiotensin II to angiotensin (1–7), which counteracts the vasoconstrictive, proliferative and inflammatory effects of angiotensin II, probably by acting through its G-protein coupled receptor Mas, also characterized by estrogen-dependent expression [34]. Moreover, since ACE2 is highly expressed in the lungs, where it also promotes immunomodulation, higher levels of ACE2 in females endow them with a protective effect against lung injury [35] and this protection may be partially imputable to endothelial barrier stabilization through the ACE2/angiotensin (1–7)/Mas axis [34]. 

Another key difference between the two sexes mediated by hormones is related to the functions of the immune system. Estrogens, by promoting both innate and adaptive immune responses, facilitate pathogens removal from the organism in females. Moreover, females show attenuated symptoms and a stronger response to vaccines. Conversely, testosterone suppresses immune function and counteract the pathways affected by estrogens, this leading to an increased susceptibility to infectious diseases in males [36,37]. Females show a higher number and activity of innate immune cells, including monocytes, macrophages and dendritic cells, compared to males, and this determines faster intervention against pathogens [35]. A more robust inflammatory response derives from the sex-specific activation of T lymphocytes in the early stage of SARS-CoV-2 infection, which is stronger in females than males, even in old age [36]. This would explain why older men are the ones with the highest risk of presenting COVID-19. Estrogens also regulate the expression and the secretion of pro-inflammatory cytokines, such as IL-1, IL10 and IFN-γ, which modulate the immune response, making it more efficient but less detrimental for the female organism [38]. Furthermore, also some small RNAs involved in the modulation of the expression of coreceptors that, together with ACE2, mediate the entry of coronaviruses (e.g., TMPRSS2, ADAM17, Furin) are regulated by sex hormones [33].

The influence of sex hormones is not limited to the susceptibility to infection or to the extent of the viral load, but it also determines the severity of symptoms and the correlated complications, thus impacting the sex-related outcomes of COVID-19 disease. By virtue of the protective role of female sex hormones, the lethality rate for COVID-19 is reduced in women of fertile age and it is noticeably lower than in men, but it gradually increases to over 20% during the post-menopausal age [39]. Premenopausal women had lower rates and short duration of hospitalization along with less requirement for respiratory support compared to post-menopausal women [39]. Moreover, a direct correlation between COVID-19 and menopausal condition and an inverse association with combined oral contraceptive pill use have been reported [40].

### 2.3. Behavior

Aside from biological sex-based differences, the gender, which is the ensemble of socially constructed characteristics of a person, may account for the gap between men and women in COVID-19 incidence and outcomes [36,41]. Indeed, women show a higher degree of COVID-19 awareness and they are more compliant to restrictive and preventive measures compared to men. Conversely, men are more prone to high-risk behaviors, such as smoking and alcohol consumption, and they often work in environments and contests with increased risk of viral exposure [41]. Additionally, men seem to follow hygiene practices with less attention and consistency compared to women, and by neglecting simple habits such as handwashing, they facilitate the infection and burden of the disease [42]. Moreover, men seem to care less about their health with respect to women, and they seek medical attention later, when symptoms are worse, this possibly explaining—at least in part—the higher severity of infections in men and resulting outcomes [36,41]. Gender differences have also been reported for nutritional habits and lifestyle. Studies conducted on western countries (US and EU) highlighted that women consume more fruit and vegetables, legumes and whole foods, while men tend to have more fat and protein-rich foods. Women appear to have a greater consciousness of the impact of nutrition on human health and are particularly concerned about their body image, and thus are more prone to adopt a healthier diet with respect to men [43]. Therefore, women could have a higher intake of micronutrients, phytochemicals and Mediterranean diet compounds with potential anti-COVID activity. Indeed, the role of platelet-activating factor (PAF) was suggested in the pathogenesis of COVID-19 and a healthy diet containing PAF inhibitors, such as vitamin A, vitamin C, vitamin E, vitamin D, selenium, omega-3 fatty acids and minerals, may target both inflammation and thrombosis and prevent the deleterious effects of COVID-19 [44].

### 2.4. Treatment and Vaccines

To date, sex-disaggregated data regarding COVID-19 treatments’ efficacy and safety are scarce. It can be predicted that sex differences in pharmacokinetics and adverse drug reactions, along with sex differences in the immune response to SARS-CoV-2, may determine a sex gap in the benefits and adverse effects of medications used to treat COVID-19, such as antiviral drugs and vaccines [45]. 

Indeed, preliminary data highlighted potential sex-related disparities in the efficacy and safety of COVID-19 vaccines. The results of a recent meta-analysis based on four clinical trials showed that, following vaccination, males appear to have a 33% reduction in the overall risk of developing COVID-19 compared to females [46]. Regarding vaccines safety, information extrapolated from pharmacovigilance reports point to an increased toxicity in women, but it should be considered that women are more inclined to report adverse drug reactions with respect to men [46].

## 3. COVID-19 and Cardiovascular Diseases (CVDs)

The SARS-CoV-2 pathogenic mechanism is mainly associated with upper respiratory tract illness, possibly evolving into severe acute respiratory syndrome (SARS), which represents one of the leading causes of hospitalization and death in infected patients. However, viral infection can also affect other organs and systems, especially those expressing high levels of ACE2, a protein acting as the functional receptor for the virus by virtue of its strong affinity to the Spike protein of SARS-CoV-2 [29,30]. Indeed, it is now well known that there is tight interplay between COVID-19 and cardiac injury [47,48,49,50]. The first evidence of this link was the increased vulnerability of patients with pre-existing cardiovascular comorbidities or with cardiovascular risk factors (hypertension, diabetes and obesity) to symptomatic infection [18,48,51,52]. Clinical studies also reported the onset of cardiovascular manifestations upon SARS-CoV-2 infection, including acute myocardial injury, myocarditis, arrhythmias, myocardial infarction and venous thromboembolism, in a significant percentage of hospitalized patients [53,54,55,56,57,58,59]. Moreover, the incidence of myocardial injury was higher in patients of the ICU than in non-ICU patients, suggesting a correlation between cardiac manifestations and COVID-19 severity [59]. Later in the pandemic, evidence of myocardial injury has been recognized as significantly affecting patients’ morbidity and mortality, being associated with worse outcomes [58,60,61], similarly to that which was previously observed in patients affected by other acute infections, such as pneumonia [62].

**Figure 2:**
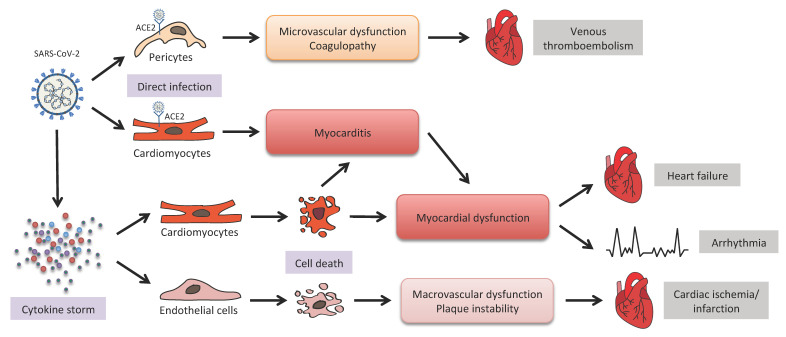
Potential mechanisms of cardiac injury in COVID-19.

A great effort has been spent to explain and predict the impact of COVID-19 on CVDs, and to clarify the mechanisms at the basis of the interplay between virus infection and the heart. In the following paragraphs we will discuss some of these mechanisms and their clinical implications, which are reported in Figure 2.

### 3.1. ACE2-Mediated Viral Infection

Several studies assessed the role of ACE2 in SARS-CoV-2 infection, demonstrating its function as a receptor for virus entry into the cell, and its downregulation upon infection as an essential negative regulatory loop to prevent further viral access. Thus, the distribution and expression of ACE2 in organs and tissues represents a key factor in determining the potential targets of SARS-CoV-2 infection. Indeed, ACE2 is enriched in the lung, which is known to be the primary affected organ of SARS-CoV-2 infection, but also in the heart, kidneys, testes, liver, intestine and brain [30]. In the context of COVID-19 cardiovascular complications, the high expression of the ACE2 protein in the heart suggests a mechanism by which SARS-CoV-2 entry could result in direct cardiac injury [63,64,65]. Such a hypothesis is further sustained by the observation of ACE2 increase after myocardial infarction, as a protective mechanism to avoid adverse cardiac remodeling [66,67,68]. Indeed, ACE2 expression on cardiomyocytes could favor SARS-CoV-2 to infect the heart, and virus-mediated downregulation of ACE2 could further contribute to cardiac injury by decreasing the ACE2-dependent compensatory protective mechanism.

However, the histopathological analysis of cardiac tissue revealed that direct cardiomyocytes infection by SARS-CoV-2 was a rare event, while most of the COVID-19-related cardiovascular presentations derived from indirect mechanisms or from pre-existing cardiac disfunctions [60,69]. The recent discovery of high ACE2 expression in pericytes in the heart opens a route to the possible infection of these cells by SARS-CoV-2 virus, with subsequent local microvascular dysfunction and coagulopathy that can contribute to the onset of venous thromboembolism, one of the most frequent cardiovascular complications in hospitalized COVID-19 patients [63]. 

### 3.2. Inflammation and Microthromboses

Indeed, the tight connection between COVID-19 and the heart should be also explained by its pathogenic mechanism, which involves the development of a robust inflammatory response. Other viral or bacterial acute infections, already known to affect the cardiovascular system, mainly act by promoting an inflammatory, prothrombotic and procoagulant state [70]. Therefore, a hyper-inflammatory state has been previously shown to contribute to CVD, through direct cytotoxic effect on cardiomyocytes, microvascular injury and intravascular coagulation, which have been described in COVID-19 pathology [71,72]. In this context, the so-called “cytokine storm” triggered by the virus may be a key mediator of cardiac damage, both directly and by the induction of endothelial cells death, which in turn may lead to increased vascular permeability and to macrovascular dysfunctions that can favor the onset of acute coronary syndromes [73]. Recently, the platelet-activating factor (PAF) has been shown to be a key molecule implicated in all these processes, since it can increase ACE2 expression, stimulate the activation of perivascular mast cells and trigger platelet aggregation [74,75]. Thus, PAF could represent one of the factors responsible for the high incidence of coronary events and thrombosis in patients with severe COVID-19 [76].

### 3.3. COVID-19 Therapies

Current clinical management of COVID-19 is mainly based on infection prevention, through control measures and an extensive vaccination campaign, and supportive care, including supplemental oxygen and mechanical ventilatory support when indicated. The spectrum of drugs to treat COVID-19 is also rapidly evolving, including antivirals (remdesivir, favipiravir), antimalarials (chloroquine, hydroxychloroquine), corticosteroids, anti-cytokine agents (IL-6 inhibitors), monoclonal antibodies and immunoglobulin therapy (convalescent plasma) [77]. Such therapies might have important side effects on the cardiovascular system and may result in cardiac toxicity. Therefore, drug administration requires caution, especially in patients who experienced cardiovascular complications. 

Antiviral drugs activity is based on the interference with viral replication, so they are mainly used in the early stages of the disease to reduce SARS-CoV-2 viral load. In clinical trials on COVID-19 patients, the nucleoside analogue ribavirin showed partial efficacy, counteracted by side effects, especially hemolytic anemia, which can contribute to increased CVD risk and hemodynamic instability [78]. The antiviral drug remdesivir, approved by the U.S. Food and Drug Administration (FDA) for the treatment of COVID-19-hospitalized patients [79], should be used with caution in patients with cardiovascular comorbidities since some cardiac adverse effects, such as bradycardia, QT prolongation and T-wave abnormality, have been observed [80,81,82,83]. 

In later stages of the disease, when the inflammatory process is more prevalent than the viral replication, the use of immunomodulatory agents can represent a valid therapeutic option [84]. Given the key role of the pro-inflammatory cytokine IL-6 in COVID-19 pathogenesis [85], some studies proposed the use of tocilizumab, a monoclonal antibody (mAb) antagonist of IL-6 receptor (IL-6R), for COVID-19 treatment [77], but its efficacy should be carefully evaluated in the light of its known side effects, such as anemia and QT interval prolongation [86]. However, other reports indicated a positive influence of tocilizumab on lipid metabolism, with a significant decrease in cardiovascular risk biomarkers [87].

Chloroquine and hydroxychloroquine, aside from their known application to treat malaria, showed good antiviral activity towards SARS-CoV-2 virus [88,89], but their use in COVID-19 should be carefully considered and monitored since their considerable cardiac toxicities led some patients to develop cardiovascular manifestations, such as QT prolongation, conduction disorders, heart failure and ventricular hypertrophy [90]. 

Recently, vaccine-associated pericarditis or myocarditis has been pointed out as a potential risk factor to be considered before vaccination [91,92]. Indeed, although mild-to-moderate cases of pericarditis and myocarditis have been reported after the second dose of the COVID-19 vaccine, especially among young adults [93,94], this adverse effect should be considered as extremely rare, and so the related risk should be considered far lower than the benefit of vaccination.

### 3.4. Long COVID

Interestingly, the development and worldwide distribution of highly effective vaccines, and the subsequent reduction in COVID-19 morbidity and mortality, shifted the interest of the medical community towards the long-term systemic effects of the disease. After acute infection, a variety of persistent symptoms have been described in COVID-19 survivors, including cardiovascular, pulmonary, neurological and mental health complications. Long-term health complications are not limited to those patients who experienced severe COVID-19, but can be observed also in patients with asymptomatic, mild or moderate acute COVID-19, characterizing the “long-hauler syndrome”, the so-called “long COVID” [10]. Among cardiovascular symptoms, the most common persistent signs among long-hauler patients are chest pain and heart palpitations, potentially leading to arrhythmias in a significant percentage of survivors of non-severe COVID-19 [95]. In patients who experienced cardiovascular events during acute COVID-19, long-term sequelae should be expected, such as systolic dysfunction, heart failure and cardiac arrhythmias, which could also require ablation procedures or cardioverter–defibrillators implant [96].

The long-term impact of the COVID-19 pandemic can also be envisioned to depend on social aspects, such as reduced access to diagnostics and deferral of routine procedures, as well as social distancing, which has been recognized as a major cardiovascular risk factor [97,98,99]. In this light, the efforts of clinicians should be oriented to identify potential long-term cardiac aftermaths, by recommending regular follow-up of cardiovascular risk assessment for COVID-19 survivors regardless of the severity of infection.

## 4. Sex and Gender Factors in COVID-19-Related CVDs

In addition to the age factor, which represents a clear element of fragility in the care context of COVID-19, the presence of different comorbidities, mainly involving the cardiovascular, pulmonary, endocrine and immunological systems, offers the possibility of identifying the categories of subjects at increased risk of severe disease and mortality, especially among patients in the age groups below 80 years old. Moreover, epidemiological and clinical studies have shown how, in the context of comorbidities, sex and gender differences represent additive biological and lifestyle variables of vulnerability to COVID-19 by altering the incidence of unfavorable and fatal outcomes [100]. Male patients show a wide susceptibility to COVID-19 pathology, having more frequent hospitalization in ICUs and a lethality rate that is about twice as high compared to women [101]. One of the main underlying reasons of this different sex-related incidence of COVID-19 progress and lethality is likely due to cardiovascular comorbidities, pre-existing or arising after SARS-CoV-2 infection. However, data on the prognostic role of cardiovascular adverse events in gender-stratified COVID-19 patients currently have yet to be fully clarified, due to the limited availability of studies or contrasting observations obtained in male and female affected individuals. Sex/gender discrepancy in COVID-19-related cardiovascular comorbidities is of fundamental importance because only by taking into consideration the complexity of biological and psychosocial factors will researchers and clinicians be able to identify strategies for reducing SARS-CoV-2 symptoms, as well as implementing adequate diagnostic–therapeutic measures, also tailored to sex differences, to counteract the progression of the COVID-19 pathology and its fatality rate.

### 4.1. Pre-Existing Cardiovascular Pathologies

Considering the comorbidities present before COVID-19 pathology, it is not yet clear to what extent these cause predisposition to SARS-CoV-2 infection, although it has certainly proved that they negatively affect susceptibility to infection and above all else, the course and severity of the disease itself. Specifically, both patients with classic cardiovascular risk factors (diabetes mellitus, arterial hypertension, obesity, old age) and patients with established cardiovascular diseases constitute a vulnerable COVID-19 population in terms of greater morbidity and mortality [102,103]. The presence of a cardiac chronic disease, such as heart failure, increases susceptibility to lung infections and flu, probably due to the reduced immune response in these patients. Many cardiovascular diseases and associated risk factors show different prevalence among male and female individuals matched for age and in a premenopausal state, due not only to biological variables (hormones, sex chromosomes, immunological response), but also to social and lifestyle factors (smoking, alcohol, high-fat diet). Thus, this sex-related incidence of cardiovascular pathologies can likely explain the more severe symptoms, worse outcomes and increased lethality rates exhibited by male COVID-19 patients compared to women. Different CVDs occur more commonly in men than in women, such as occlusive coronary heart disease, stroke, carotid stenosis and cardiac hypertrophy [104], with classical risk factors having a different gender-related impact for CVDs. In particular, aging, hypertension, cholesterol levels, overweight and obesity have a more effective correlation with CVDs in men than in women [104], who also pay more attention to healthy eating [105] and healthcare services. One of the biological mechanisms known to be responsible for these cardiovascular events is the continuous development of the atherosclerotic process in men, whilst atheroprotective effects are exerted in women by estrogens, which prevent endothelial dysfunction and control cardiac lipid accumulation by lowering LDL and raising HDL cholesterol levels. In this context, many studies have well established the adverse impact of testosterone on the molecular pathways and epigenetic mechanisms involved in cardiac inflammation, arteriosclerotic plaque formation, platelet aggregability, heart rhythmicity and hypertrophy [106,107], whilst female hormones have a protective effect on the cardiovascular system by reducing heart fibrosis and hypertrophy, by counteracting cardiomyocyte apoptosis and finely regulating inflammatory response to cardiac insults [106,107]. Thus, it is not surprising that the decrease in estrogen levels in women of post-menopausal age leads to a greater risk of cardiovascular pathologies and, in turn, to a higher possibility to develop a severe form of COVID-19 with respect to women of childbearing age and to age-matched men. However, females still show reduced mortality related to COVID-19, and this may partially be ascribed to sex-related mechanisms, such as an improved reparative response following cardiovascular injury [36].

### 4.2. COVID-19 Cardiovascular Complications

COVID-19 is primarily a respiratory disease, but many patients have experienced cardiovascular complications, such as acute heart damage, myocarditis, stress cardiomyopathy, myocardial infarction, arrhythmia and thromboembolic events arising after SARS-CoV-2 infection (Figure 2) [108]. These complications can have short- and long-term consequences that can also increase the risk of mortality. Indeed, raised levels of myocardial damage biomarkers have been observed in many patients with COVID-19 disease [109], this correlating with a more severe course and higher mortality. Cardiovascular complications may be consequent to myocardial damage, secondary to respiratory failure, which in turn determines hypoxia, hypotension and increases the cardiac workload, and/or myocarditis, or fulminant. This is due to direct viral infection or the cytokine storm, typical of severe infections, that causes the activation of cells within pre-existing atherosclerotic lesions, increasing the risk of thrombosis and therefore the risk of myocardial ischemia (Figure 2) [110]. Incidence of cardiovascular complications in COVID-19 pathology appear to be associated to sex and gender differences, this likely being the reason for the greater severity and poorer outcomes of the SARS-CoV2-mediated disease in male patients compared to women [111]. To date, there are already very few and controversial data stratified by sex allowing us to understand the role of cardiovascular complications in the different prognosis and outcome of COVID-19 disease in men and women. Women have been shown to have increased expression and activation of the angiotensin type 2 (AT2) receptors, which are not only involved into a more robust anti-inflammatory immune response against SARS-CoV-2 infection, but are also implicated in the regulation of blood pressure and renal function, thereby providing protection for cardiovascular complications in female patients [112]. In particular, dysregulation of the RAS is known to play a major role in the progression of cardiovascular disease in humans, due to the production of angiotensin II that promotes vasoconstriction, inflammation and deleterious cardiovascular effects. ACE2 normally acts to counterbalance the RAS by degrading angiotensin II. SARS-CoV-2 infection, decreasing the levels of bioavailable ACE2 through competitive inhibition of the enzyme binding site or through cleavage of the active site ectodomain [113], reduces its protective action on the RAS, thus favoring cardiovascular damage. Thus, the degree of downregulation of ACE2 is associated with the severity of disease. Estrogen-dependent higher levels of ACE2 and enhanced activity of the ACE2 pathway most likely contribute to the reduced incidence of cardiovascular disease in premenopausal women [114]. Looking at the various heart-related adverse events, the different incidence of myocardial injury in observational studies disaggregated by sex remains controversial, with some studies highlighting more prominence in men than in women [60,115], whilst others report no significant sex-dependent differences [58]. Notably, the degree of cardiac cell death is more pronounced in males than females under several conditions [111], and this may be related to an enhanced repair response in females with respect to males [116]. Indeed, females produce high levels of reparative leukocytes and epoxy-eicosatrienoic acids with antihypertensive and anti-inflammatory effects on blood vessels [116], and this determines limited cardiac remodeling and more efficient functional recovery [24]. 

Cases of COVID-19-related myocarditis have been described, but it seems that a host immune response can occur more frequently, triggering a systemic inflammatory syndrome with endothelial damage involving the myocardium itself [61]. Myocardial injury has been reported to be due to the direct infection of the heart by SARS-CoV-2 viral particles through binding to ACE2 receptors, which are also expressed by several cardiac cell types. To date, there are no epidemiological data divided by sex on the prevalence of myocarditis induced by SARS-CoV-2 infection but, starting from the evidence that sex hormones are able to differently control the expression of ACE2 receptor genes [83], it is likely that sex differences may exist in the onset of inflammation of the heart muscle. Overexpression of inflammation proteins, such as IL-1β and IL-6, triggered by SARS-CoV-2 infection, appears to be associated with cardiovascular events. Many of the epidemiologic data come from the Chinese population, where it was observed that dead patients had heart damage, coronary artery disease, heart failure and cerebrovascular disease [58]. 

Acute coronary syndrome in the absence of coronary lesions (MINOCA), characterized by the onset of chest pain, elevation of Troponin-HS and abnormalities of parietal kinesis, was confirmed to have a higher prevalence in women with symptoms of SARS-CoV-2 infection [117]. Acute coronary syndrome induced by COVID-19 seems to involve systemic inflammatory events, such as the activation of macrophages releasing collagenases and MMPs that lead to plaque rupture, as well as a sustained cytokine storm or microthrombus formation.

The pathogenesis of COVID-19 induces alterations of the coagulation system, by promoting inflammation and endotheliopathy, which in turn leads to the formation of disseminated thrombi, worsening the greater thromboembolic risk of men compared to women [42]. Indeed, venous and arterial thromboembolic complications, observed in several organs of patients with SARS-CoV-2 virus, are likely linked to events of thrombosis in alveolar and pulmonary capillaries derived from the abnormal activation of the coagulation cascade. Available data on the incidence of thrombotic complications in patients infected with SARS-CoV-2 are already too small to assert significant gender-related differences; however, recent studies highlighted a trend of higher incidence of venous and arterial thromboembolic events in hospitalized male COVID-19 patients compared to affected women [118,119]. In standard conditions of exposure to normal levels of sex hormones, the risk of thromboembolism is at least threefold higher in men than in women at any age, with a frequency that is higher in men during elderly age and lower in women throughout fertile age [120]. Female patients using hormonal contraception represent a risk category for venous thromboembolic disease [121,122], this being related to inappropriate activation of the coagulation cascade likely because of the pro-inflammatory cytokine storm induced by SARS-CoV-2 viral infection. 

To date, no significant differences have been observed in the proportion of men and women with COVID-19 presenting complications of cardiac arrhythmias [123]. Indeed, atrial fibrillation, sinus tachycardia and bradycardia, prolonged QT, as well as cardiac arrest have been shown to be associated with COVID-19 pathology, but whether sex differences exist is important to understand. 

Concerning cardiovascular complications secondary to COVID-19 therapies, there is limited information about sex differences, mainly due to the prevalent inclusion of men in randomized clinical trials and to the lack of sex-targeted treatment strategies [39]. Tentative considerations could be made about antiviral therapies, due to the previous knowledge of differential efficacy and safety of antiviral drugs between sexes, with increased adverse effects in females, likely due to differences in the pharmacokinetics and pharmacodynamics profiles and to the influence of sex hormones [124]. As for COVID-19, the only sex-related information is the increased probability of men to receive antiviral therapy, probably related to the higher vulnerability and incidence of comorbidities [125,126]. 

To date, little information about sex differences in long COVID sequelae are available. A higher incidence of headaches, abdominal symptoms and psychological complaints has been reported in females, whereas males were more prone to breathing difficulties and cognitive symptoms [127]. No data are still available specifically regarding the impact of sex/gender in long-term cardiac complications.

## 5. Sex-Related Biomarkers of COVID-19 Cardiovascular Complications

As a systemic infection, SARS-CoV-2 causes an inflammatory response that induces cellular and biochemical modifications in the composition of peripheral blood [128]. A significant number of patients have reported exhibiting a plethora of alterations in the gastrointestinal tract, kidneys, heart and liver, to mention a few [129,130,131,132]. Although not restricted to the pulmonary area, these alterations often result in abnormalities in the levels of circulating biomarkers such as bilirubin, creatinine and coagulation factors. As discussed above, biological differences, gender-specific behavioral factors and pre-existing rates of comorbidities result in a combination of elements that might explain the disparity between men and women’s mortality. In this context, it will be important to characterize the proposed molecular and cellular markers of COVID-19 infection based on their sex-related differences, to better assess the effect of patient sex on the relationship between biomarkers and COVID-19 outcomes [133,134].

### 5.1. Cardiac Biomarkers

Given the severe cardiovascular implications of COVID-19 infections, cardiac biomarkers are considered valuable aids to determine the severity and predict the course of the infection. Indeed, these biomarkers allow us to detect and quantify the impact of COVID-19 infection on the cardiovascular system through clinical predictors such as cardiomyocyte injury, quantified by cardiac troponin (cTn) concentrations, and hemodynamic cardiac stress, quantified by natriuretic peptide concentrations [135,136]. The level of those biomarkers correlates with disease severity and mortality and may aid in risk stratification of COVID-19 patients. In particular, some reports indicated a higher level of cTn in approximately one-third of COVID-19 patients, with a significant increase in older people and in males vs. females [60,137]. Moreover, increases in cTn have been observed in patients with severe COVID-19 presentations, being associated with adverse outcomes and higher risk of death. Thus, the evaluation of this marker in hospitalized patients might facilitate risk stratification and help make decisions about the therapeutic strategies to adopt [138].

Concerning natriuretic peptides (NPs), mainly B-type natriuretic peptide (BNP) and NTproBNP, they might be elevated in COVID-19 patients both due to the presence of pre-existing cardiac disease and the occurrence of acute hemodynamic stress related to COVID-19 pathogenesis [139]. Elevated levels of NPs have been observed in older patients and are positively associated with patients’ mortality [137]. No data are available about sex differences in NPs levels. Indeed, the scientific community is still debating about the opportunity to include NPs measurement in the COVID-19 diagnostic panel, since the increase in these markers still seems to provide limited prognostic information. 

### 5.2. Thrombotic Markers

With respect to coagulation factors, a significant increase in activated partial thromboplastin time (aPTT), prothrombin time (PT/INR) and D-dimer was observed in COVID-19 patients [140]. Such evidence reflects the activation of the hemostatic system upon SARS-CoV-2 infection and shows a positive correlation with COVID-19-related thrombotic manifestations, such as microvascular coagulopathy, venous thromboembolism and pulmonary embolism. It is interesting to note that differences have been highlighted between men and women regarding the timing of restoration of these biomarkers. In fact, considering COVID-19 patients in the ICU, aPTT levels recovered within one month in men with severe infection, whilst this recovery time was not observed in women. A similar trend was also observed for D-dimer levels [140]. D-dimer might also be used as death predictor in patients developing thrombosis with thrombocytopenia syndrome following COVID-19 vaccination. In fact, among the risk factors for the developing of this syndrome we can find female gender, comorbidities and elevated D-dimer levels [141].

### 5.3. Inflammatory Response Markers

The central role of inflammation in virus-mediated myocardial injury is sustained by increased concentrations of plasmatic cytokines, such as interleukin-6 (IL-6), in COVID-19 patients with cardiac injury [109] and by the clinical evidence of an association between the cardiac damage marker cTn and known inflammatory markers, including C-reactive protein (CRP), procalcitonin (PCT), ferritin and fibrinogen [58,63]. Sex-related differences in the levels of inflammatory markers have also been reported in COVID-19 patients [142,143,144]. A recent analysis showed that CRP, IL-6, PCT and ferritin are significantly higher in men compared to women [145]. Risitano et al. [146] also observed increased levels of markers such as CRP, ferritin, LDH, fibrinogen and GGT (gamma-glutamyltrasferase) in older men with COVID-19. The increased inflammatory response due to the higher values of the aforementioned markers, as a consequence of an increased production of pro-inflammatory cytokines and complement activation, might also negatively influence patients’ prognosis. Therefore, substantial sex differences in the production of inflammatory molecules might be heavily involved in regulating COVID-19 clinical outcome, as is already known for several other types of respiratory tract viruses, such as SARS-CoV-1 or MERS, in which sex hormones cause a different immune response [147,148]. In fact, the higher production of inflammatory markers in men is accompanied by an increased risk of death from COVID-19 infection, and the correlation between inflammatory markers (i.e., CRP) and death or ICU admission is more evident in men than in women [145].

### 5.4. Serum Renin

As previously discussed, CVDs can lead to a higher risk of developing COVID-19 and could also cause higher COVID-19-associated mortality [149,150]. It is widely known that cardiovascular comorbidities, old age and male sex are linked by a relative deficiency of ACE2 [31,151,152]. Given the role of ACE2 in the RAS signaling, the difference between young and old and between men and women in ACE2 expression could lead to age- and sex-related differences in renin levels [153]. Indeed, circulating renin levels have been shown to increase with age, and to be higher in men than in women since around puberty [154]. Hormonal regulation may have a role in these differences, as sex hormones have effects on immune cell function [155,156]. Indeed, the correlation between renin levels and puberty might uncover an underlying connection with increased testosterone levels, as demonstrated in murine models by White et al. [157]. Similarly, the inversed trend of circulating renin levels in women compared to men could be explained by downregulation of renin by estrogens. Thus, circulating renin levels are positively correlated with soluble ACE2 levels, which are higher in males compared to females [154,158,159]. With these premises, it is then reasonable to speculate a correlation between the shedding of membrane-bound ACE2 and increased RAS-signaling activity in men compared to women, thus indicating a positive correlation between higher renin levels and increased risk of developing severe COVID-19.

## 6. Conclusions

The present review summarizes evidence of the profound impact of sex and gender diversity in the COVID-19 pandemic. 

The implications of sex and gender on the status of health and disease represent an area of great interest in the clinical management of patients. Indeed, differences between men and women in health terms are not attributable exclusively to biological and reproductive differences and specificities, which are determined by sex, but also to environmental, social, cultural and relational factors, which are determined by gender. 

**Figure 3:**
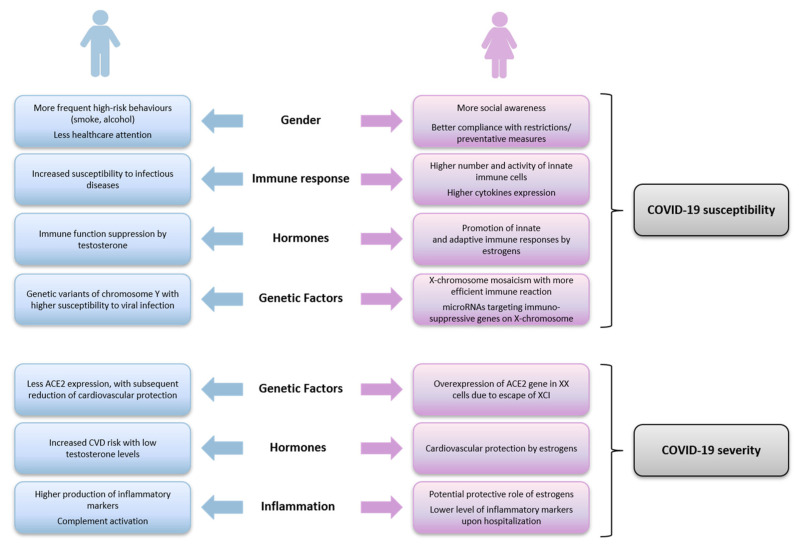
Schematic diagram of sex and gender factors potentially involved in the differential COVID-19 susceptibility and severity between males and females.

As reported in Figure 3, we discussed the principal factors potentially involved in the differential susceptibility and outcome of COVID-19 between men and women. Among them, we identified gender differences, mainly related to high-risk behaviors, nutrition and healthcare attention, as well as sex differences, involving genetic factors, influence of sexual hormones, variabilities in the function of immune system and in the regulation of inflammatory response.

Men and women may have very different pathological symptoms as well as responses to drugs with significant impact in the field of research, prevention, diagnosis and treatment. This is particularly true in COVID-19, where sex and gender differences are reflected in significant variations in ACE2 gene expression, immune response and the pathophysiology of cardiovascular risk factors and comorbidities, contributing to lower susceptibility to infection and better survival in women and, conversely, relatively higher hospitalization and mortality rates in men. 

Further studies are required to provide a better understanding of sex and gender influence in the reaction to various treatment options, or in the long-term sequelae of COVID-19 infection, with the aim of setting up personalized prevention programs, clinical evaluation and therapies for a more efficient resolution of the disease.

## Data Availability

The data analyzed in this study are available online: https://globalhealth5050.org/the-sex-gender-and-COVID-19-project/the-data-tracker/?explore=variable&variable=Confirmed+cases (accessed on 2 November 2021).
